# Pharmacovigilance of antimalarial treatment in Africa: is it possible?

**DOI:** 10.1186/1475-2875-5-50

**Published:** 2006-06-16

**Authors:** Ambrose O Talisuna, Sarah G Staedke, Umberto D'Alessandro

**Affiliations:** 1 Ministry of Health Epidemiological Surveillance Division, Po Box 7272, Kampala, Uganda; 2 Prince Leopold Institute of Tropical Medicine, Nationalestraat 155, B-2000, Antwerp, Belgium; 3 Department of Medicine, San Francisco General Hospital, University of California, San Francisco, USA

## Abstract

Pharmacovigilance, defined as "the science and activities relating to the detection, assessment, understanding and prevention of adverse effects or any other possible drug related problem", is increasingly being recognized in Africa. Many African countries have simultaneously adopted artemisinin derivative based combination therapy (ACT) as first-line treatment for uncomplicated malaria, offering an opportunity to assess the safety of these drugs when used widely. While ACTs appear to be safe and well-tolerated, there is little experience with these medicines in Africa, outside clinical trials.

Pharmacovigilance for ACTs and other combination treatments in Africa is essential. Malaria transmission intensity is high and antimalarial medicines are used frequently. Presumptive treatment of fever with antimalarials is common, often in the absence of a confirmed diagnosis, using drugs obtained without a prescription. Informal use of antimalarial drugs may increase the risk of incorrect dosing, inappropriate treatment, and drug interactions, which may impact negatively on drug safety. Furthermore, the administration of antimalarial treatments in patients with a concomitant illness, including HIV/AIDs, tuberculosis and malnutrition, is a concern.

African countries are being encouraged to establish pharmacovigilance systems as ACTs are rolled out. However, pharmacovigilance is difficult, even in countries with a well-developed health care system. The rationale for pharmacovigilance of antimalarial drugs is discussed here, outlining the practical challenges and proposing approaches that could be adopted in Africa.

## Background

**Figure 1:**
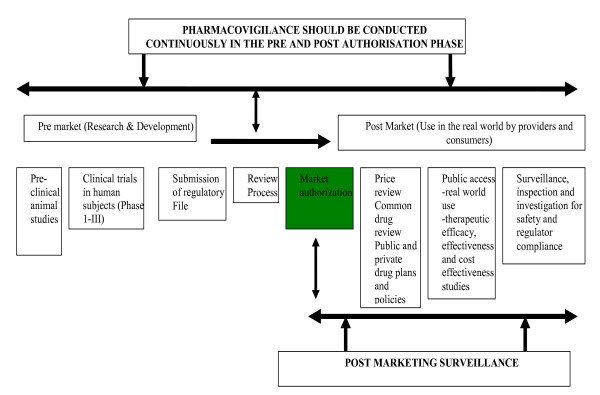
Scheme for research, development, access and use of medicinal products and the timing of pharmacovigilance and post marketing surveillance.

Prior to product registration and marketing, data about the safety and efficacy of drugs are limited to observations from pre-clinical animal studies and initial clinical trials (Phase I-III). This is the basis for the summary of product characteristics or the label of the product. Although such trials are useful for product registration, they typically evaluate only a small number of specifically selected participants under ideal conditions and have limited statistical power to detect the uncommon side effects. As a result, clinical trials and the information that is derived from them are inadequate for the full assessment of safety risks. Consequently, pharmacovigilance (PV), conducted continuously from the time a drug is developed to the post-marketing phase, becomes critical in evaluating safety risks. Pharmacovigilance has been defined as "the science and activities relating to the detection, assessment, understanding and prevention of adverse effects or any other possible drug-related problem" [1]. The term "post-marketing surveillance" (PMS) is sometimes interchangeably used with PV. However, PV and PMS differ in the features evaluated and in the timing of the surveillance (Figure 1). PV primarily focuses on the assessment of safety risks and begins in the pre-market phase, continuing after the product is released onto the market. PMS involves monitoring the quality, efficacy and safety of registered products that are already available on the market.

### Rationale for pharmacovigilance of antimalarial drugs

Interest in establishing systems for pharmacovigilance of antimalarial drugs in Africa is gaining momentum [2]. In response to widespread resistance to older antimalarial drugs, many African countries have adopted combination therapy (generally ACT) as first-line therapy for treatment of uncomplicated malaria, and are planning to use these new regimens on a wide scale in the public and private sector. ACTs have been used successfully in Southeast Asia and appear to be safe and well-tolerated [3-5]. However, there is relatively little experience with these drugs in Africa, where malaria transmission intensity is substantially greater and the pattern of antimalarial drug use is quite different. In areas of intense transmission, antimalarial drugs are given repeatedly to treat frequent fevers (even in the absence of malaria). In addition, the use of antimalarial drugs outside the formal health sector, often for presumptive treatment of fever, is a widespread practice in Africa [6-9]. In Uganda, for example, a study conducted at seven sites around the country found chloroquine metabolites in the urine of 32–80% of the general population [10]. Antimalarials are often purchased from drug shops or pharmacies without consulting a health care worker. Such informal use of antimalarials could increase the risk of incorrect dosing, inappropriate treatment and interactions of different medicines, which could have a negative impact on antimalarial treatment safety. In addition, the safety of antimalarial treatment in vulnerable populations including pregnant women, and in patients with coexisting illnesses (such as HIV/AIDs, tuberculosis and malnutrition) has not yet been established. The current plans for wide-scale implementation of ACTs across Africa offer an important opportunity to evaluate the safety of medicines. Identification of rare and unexpected adverse events and evaluation of antimalarial treatment safety in early pregnancy are high priorities for PV of ACTs. However, PV is challenging, even in countries with well-developed health care systems. To develop practical and sustainable PV systems in Africa, innovative solutions will be required.

### Practical challenges for establishing pharmacovigilance systems in Africa

The critical definitions in pharmacovigilance are summarized in Table 1[11]. It is important to recognize the distinction between an "adverse event (AE)" and an "adverse drug reaction" (ADR). While an adverse event is any undesirable medical occurrence that develops after the administration of a drug, regardless of the suspected relationship between the drug product and the event, to classify an event as an adverse drug reaction, a causal relationship must be established. These definitions highlight some of the practical challenges of establishing a PV system for antimalarial treatment in Africa, related to the detection of adverse events and the determination of the severity and relationship of events to a specific product. Health workers at the point of data collection are required to detect adverse events, which are often difficult to distinguish from common symptoms of malaria. Once an adverse event has been detected, the maximum severity of the event should be established. Standardized guidelines have been provided by the World Health Organisation (Table 2) and other organizations [11], but the grading may be subjective. Classification of the relationship of an adverse event to a product is another challenge (Table 3). Determining if an event has been caused by a given product or is related to other concomitantly administered drugs, malaria, or other illnesses, is also difficult and often subjective. In addition, defining the period of "reasonable temporal association" between an event and prior treatment is problematic when considering combination therapies that include partner drugs with long elimination half-lives. Assigning a causal relationship and determining if an event is unexpected is even more difficult when multiple drugs have been administered together.

**Table 1:** Important definitions for PV (ICH guidelines [11])

**ADVERSE EVENT**
Any untoward medical occurrence in a patient or clinical investigation subject administered a pharmaceutical product and which does not necessarily have a causal relationship with this treatment. An AE can therefore be any unfavourable and unintended sign (that could include a clinically significant abnormal laboratory finding), symptom or disease temporally associated with the use of a medicinal product, whether or not considered related to the medicinal product (ICH guidelines).

**ADVERSE DRUG REACTION**

Any noxious and unintended responses to a medicinal product related to any dose. The phrase "responses to a medicinal product" implies that a causal relationship between a medicinal product and an adverse event (AE) is at least a reasonable possibility (i.e. the relationship can not be ruled out).

**UNEXPECTED ADVERSE DRUG REACTION**

An adverse reaction, the nature or severity of which is not consistent with the applicable product information (e.g. investigators brochure for an unapproved product or package insert/summary of product characteristics for an approved product (ICH guidelines).

**SERIOUS ADVERSE EVENT**

Any untoward medical occurrence that at any dose: results in death; is life-threatening; requires hospitalization (other than for drug administration) or prolongation of existing hospitalization; results in persistent or significant disability/incapacity; or is a congenital anomaly/birth defect and also other important medical events that jeopardise the subject or require intervention to prevent one of the other outcomes listed in the definition above).

**Table 2:** Guidelines for grading severity of adverse events

**Grade**	**Severity**	**Description**
1	Mild	Awareness of sign or symptom, but easily tolerated: Transient or mild discomfort; no limitation in activity; no medical intervention/therapy required
2	Moderate	Discomfort enough to cause interference with usual activity: some assistance may be needed; no or minimal medical intervention required
3	Severe	Incapacitating with inability to work or perform usual activity: some assistance usually required; hospitalization possible
4	Life-threatening	Extreme limitation in activity, significant assistance required; significant medical intervention/therapy required; hospitalization probable

**Table 3:** Relationship of adverse events to antimalarial agents

**Classification**	**Definition**
Definitely related	Events have no uncertainty in their relationship to test drug administration: meaning that a re-challenge was positive.
Probable	The suspected adverse event follows a reasonable temporal sequence from study drug administration, abates upon discontinuation of the drug, and cannot be reasonably explained by the known characteristics of the subject's clinical state.
Possible	The suspected adverse event may or may not follow a reasonable temporal sequence from study drug administration but seems to be the type of reaction that cannot be dismissed as unlikely. The event could have been produced or mimicked by the subject's clinical state or by other modes of therapy concomitantly administered to the subject.
Unlikely	There is no reasonable temporal association between the study drug and the suspected event and the event could have been produced by the subject's clinical state or other modes of therapy administered to the subject.
Definitely unrelated	Events which occur prior to test drug administration or for those events which cannot be even remotely related to study participation (e.g. injury caused by a third party).

Other challenges related to the reporting procedures include: What events should be reported? All adverse events or only adverse drug reactions? Should all events be reported irrespective of severity grade? Or should only more severe events be reported? Should the relationship and expectedness of events impact on reporting? African national malaria control programmes (NMCPs) and national drug regulatory authorities will have to address such questions before PV for antimalarial treatments can be established.

### Application of pharmacovigilance (PV) methods in Africa

Two broad approaches for pharmacovigilance are used in developed countries, including passive spontaneous reporting systems, and systems utilising pharmaco-epidemiological methods. Both methods could be applied in Africa depending on the circumstances. Potential approaches of utilizing these methods in Africa are suggested here.

### Passive follow-up of marketed products post-authorisation

Generally, post-authorisation follow-up is done using a passive approach with continuous reporting of adverse events and re-evaluation of the risks and benefits of drugs. Advantages of a passive reporting system include low cost, simplicity and the ability to detect rare events and continuously monitor safety. However, passive reporting systems are currently limited or non-existent in most countries in sub-Saharan Africa, but they could be strengthened (or developed) by focusing on designated sentinel health facilities such as district hospitals, and targeted populations such as pregnant women and children less than five years of age. Potential challenges should be recognized at the outset including the risk of under-reporting, and the difficulty of establishing a causal relationship between adverse events and specific drugs. A routine PV system for antimalarial treatments in Africa could be initially targeted to capture data on severe events, and those deemed to be related to antimalarial drugs. Establishing a system for passive post-authorisation follow-up as a basis for signal detection should be a long term commitment of national governments in Africa. The national drug regulatory agencies should work closely with the national malaria control programmes and other relevant programmes such as AIDS control programmes to provide stewardship. Multi-disciplinary national PV centres should be established and their capacity should be strengthened for regular analysis of safety data to identify and review signals, generate hypotheses, and to ensure regular reporting and feedback.

### Pharmacoepidemiological methods

#### Post-licensure clinical trials to assess effectiveness and safety of medicines in the "real world"

Phase IV studies, unlike Phase III clinical trials aim to evaluate the performance of drugs in a "real world" setting, with minimal interference. A number of post-licensure antimalarial drug efficacy trials conducted in Africa have assessed safety, suggesting that Phase IV studies are a feasible approach for PV in an African setting [12-17]. However, in order to adequately assess safety risk in such studies, the sample size estimation should ideally be based on a primary safety outcome, rather than efficacy or effectiveness, which may necessitate evaluation of a large number of patients in multiple settings. The main practical challenges for monitoring safety in such studies are likely to be similar to those encountered in Phase III clinical trials – specifically, how to differentiate events related to antimalarial treatments from symptoms of malaria or other illnesses, and how to establish the severity, relationship and expectedness of adverse events. In Phase III clinical trials, comparison of multiple treatment groups is used to test hypotheses that efficacy and safety varies with antimalarial treatment. This approach could also be applied to Phase IV studies, if the sample size is adequate to assess risks in multiple arm treatments, and if the collection of safety data is standardized to ensure high quality. In the past, the need for pharmacovigilance was less recognized and antimalarial drugs were simply released on the market, with little PV and PMS. The suggestion that newer antimalarial drugs be monitored closely after registration is not meant to imply that these drugs are more harmful than older drugs. Post-licensure clinical trials are important and should be supported by the pharmaceutical industry, working in close collaboration with researchers and academia. Indeed in developed countries, PV is a legal obligation for the pharmaceutical industry. Pharmaceutical companies should also support African countries to conduct post-licensure surveillance, focusing on safety risks for newer products, as part of their social responsibility.

### Case control studies

Case-control studies could be used to retrospectively evaluate newly detected ADRs and assess the risk of adverse events among patients exposed to a given drug. Such studies are useful for evaluating rare events and can frequently be conducted quickly, using a small sample size, at relatively low cost. Case control studies are likely to be suitable for evaluating the risk of adverse events in pregnant mothers who are inadvertently exposed to antimalarial treatments in the first trimester. However, the events must first be detected before case control studies can be conducted. Establishing and maintaining detailed antenatal and pregnancy registers to collect good quality data on exposure to medicines during all the trimesters of pregnancy will facilitate detection of potential adverse effects. Several African countries currently maintain antenatal registers as part of their routine health service data collection systems and such registers should be revised to capture data on drug safety. In addition, the collection of data on antimalarial drug exposure during pregnancy should be a mandatory requirement for the national health management information systems. At delivery, at the time of routine vaccination or at any point of contact with the health care system during the first year after delivery, a follow-up assessment of serious adverse events could then be conducted.

### Active population-based evaluation of marketed products post-authorization

Prospective studies of a reasonably sized cohort exposed to treatments and assessed for any subsequent AEs could be used to monitor relatively common events. Active surveillance requires prospective follow-up of a given population to determine the incidence of adverse events in those exposed to a given medicine. This approach is probably the most ideal, yet least pragmatic of all the pharmaco-epidemiological methods because it is very expensive and laborious. A major challenge for prospective cohort studies will be the identification of treated (exposed) patients. However, in areas where there are well-established prospective demographic surveillance sites (DSS) in Africa (see the IN-DEPTH network), a PV system could be linked to the DSS sites. A sample of the population under surveillance (ideally children less than five years old and pregnant women) would be selected and followed for an extended period (1–3 years). Monitoring for adverse events would begin at the time of first antimalarial treatment, with periodic follow-up of participants for 1–2 months following each antimalarial treatment. Where DSS systems are lacking, communities living within the catchment areas for the sentinel sites used for monitoring antimalarial treatment within the framework of the East African Network for Monitoring Antimalaria Treatment (EANMAT), the West African Network for Monitoring Antimalaria Treatment (WANMAT I and II) and the Horn of Africa Network for Monitoring Antimalaria Treatment (HANMAT) could be used to establish a targeted active population-based surveillance. In addition, in countries with a policy of home-based management of fevers such as Uganda, active surveillance for drug safety could be conducted by drug distributors. Such approaches would require substantial financial and human resource investment. However, costs could be reduced if they were embedded within the broader framework for monitoring and evaluation of the impact of malaria interventions, a key requirement to track the achievement of the millennium development goals (MDGs) [18,19].

### Is pharmacovigilance of antimalarial treatment in Africa possible?

**Figure 2:**
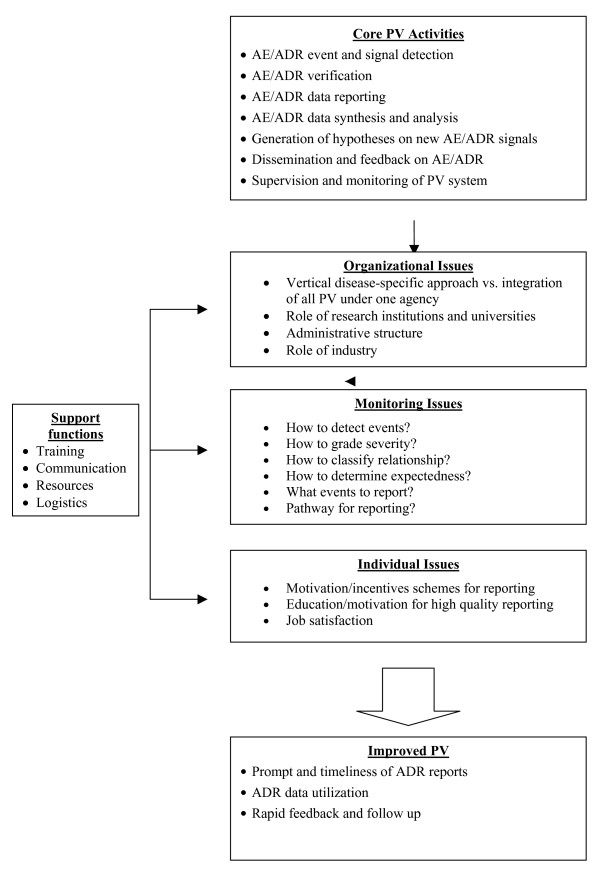
Conceptual model for strengthening pharmacovigilance in Africa modified from the model for strengthening multi disease surveillance in the WHO/African region [20].

PV for antimalarials and other drugs will be challenging in Africa. Transparency in the management of drug safety issues must be ensured. Optimal risk management cannot be guaranteed unless access to information for patients, health care providers and the community in general is improved. The World Health Organisation regional and sub-regional networks should facilitate member countries to create a database on ADRs reported in the African region for all newer antimalarial medicinal products. The level of accessibility to this database by health care professionals and the public should be defined from the outset. Country-specific proposals or strategic plans for PV should be developed by national malaria control programmes and national drug regulatory bodies focusing on needs assessment and the priority data required. In establishing sustainable pharmacovigilance systems for antimalarials in Africa, countries will need to critically assess the system that will be used for detection, evaluation of severity and evaluation of relationship of adverse events. Practical issues of how such reporting will be done, and by whom, will need to be addressed. After a system has been designed, the capacity for conducting the work will need to be strengthened or established. In order to build capacity for PV, a conceptual model developed for strengthening multi-disease surveillance at country level within the WHO region for Africa, has been adapted [20]. Based on this model, the core and the support functions for pharmacovigilance planning (PVP) and capacity strengthening, have been identified (Figure 2).

## Conclusion

Pharmacovigilance has evolved over time in developed countries. Although inadequate infrastructure and limited resources (both financial and human) pose challenges to implementing PV in developing countries, increased vulnerability of local populations makes drug safety monitoring imperative. Establishing systems for PV in Africa will be the initial step. Assessment of strengths and weaknesses, expansion of existing infrastructure, and development of programs to educate, motivate, and provide feedback will be essential to help build capacity for PV. Standardizing methods of reporting, including approaches for defining events, determining severity, and assessing causal relationships will also be important. Integrating PV for malaria with other existing systems, such as monitoring for adverse events following immunization, and newer programs to monitor safety of treatment for other diseases, including antiretroviral therapy in HIV/AIDS, will likely be needed to leverage resources and avoid duplication of efforts. Once PV systems are operational, rigorous and continuous quality assurance and conduct of replicable validation studies will be critical if PV is to be successful. Such assessments will also help to determine the best practices with respect to the frequency of safety data collection and analysis, and the optimal combination of methods for PV. PV in Africa should be possible, but realistic assessment of the challenges and introduction of novel solutions will be needed to build sustainable programs. The present momentum for PV should be harnessed to develop innovative proposals for PV in Africa [2]. Initially, monitoring activities could target antimalarial drugs, but ultimately PV should be expanded to include safety monitoring of all drugs.

## Conflict of Interest

The author(s) declare that they have no competing interests.
